# Strain-dependent release of cytokines modulated by *Lactobacillus salivarius *human isolates in an *in vitro *model

**DOI:** 10.1186/1756-0500-3-44

**Published:** 2010-02-25

**Authors:** Lorenzo Drago, Lucia Nicola, Enrico Iemoli, Giuseppe Banfi, Elena De Vecchi

**Affiliations:** 1 Laboratory of Microbiology, Department of Preclinical Sciences LITA Vialba, University of Milan, Milan, Italy; 2 Laboratory of Clinical Chemistry and Microbiology, IRCCS Galeazzi Institute, Via R. Galeazzi 4, 20161 Milan, Italy; 3 Allergology Unit, L. Sacco Teaching Hospital, Milan, Italy; 4 Scientific Direction, IRCCS Galeazzi Institute, Department of Health Technologies, University of Milan, Milan, Italy

## Abstract

**Background:**

Oral administration of probiotics is known to modulate cytokines profile not only locally, but also systemically. Four strains of *Lactobacillus salivarius*, LDR0723, BNL1059, RGS1746 and CRL1528, were evaluated for their ability to modulate release of pro- and anti-inflammatory cytokines.

**Findings:**

Strains were assessed for effects on production of Interleukin-12 (IL-12), Interferon-γ (IFN-γ), Interleukin-4 (IL-4) and Interleukin-5 (IL-5) by incubating bacterial suspensions with THP-1 macrophage like cells. Cytokines were determined by means of specific quantitative enzyme-linked immunosorbent assays.

LDR0723 and CRL1528 led to a sustained increment in production of IL-12 and IFN-γ and to a decrease in release of IL-4 and IL-5, while BNL1059 and RGS1746 favoured Th2 response, leading to a decrease in Th1/Th2 ratio with respect to unstimulated cells.

**Conclusions:**

In conclusion, capability of *L. salivarius *to modulate immune response was strictly strain dependent and strains of the same species might have opposite effects. Therefore, a careful evaluation of anti-inflammatory properties of lactobacilli should be performed on single strain, before any consideration on potential probiotic use.

## Findings

Oral administration of lactobacilli may modulate cytokines profile not only at intestinal level but also systemically [1]. The main difference between the mucosal and systemic immunities is that in the former the mechanisms of innate immunity and the activation of B cells for mucosal immunity are more important than the adaptive immune response involving the T cell population.

At the gut mucosal level, the innate immune response provides the first line of defence against pathogenic microorganisms which is initiated by immunoglobulin A (IgA) secretion. Studies with animal models have shown that intestinal microorganisms increase the numbers of IgA-secreting plasma cells, thus up-regulating IgA secretion, although the precise mechanisms underlying the way these bacteria modulate the intestinal immune system still remain unclear. Interactions among antigen-presenting cells (APCs) naïve T cells and B cells lead to generation of B cells with a high level of IgA expression. Ag-activated T and B cells may migrate from the inductive environment to effector sites through lymphatic drainage and blood-stream. Multiple cytokines, including TGF-β and IL-4, IL-5, IL-6 and IL-10 are required to promote IgA class switching and maturation [2].

Cytokines produced by immunocompetent cells such as APCs and T lymphocytes have been advocated to play a significant role in several diseases, such as inflammatory bowel disease (IBD), irritable bowel syndrome (IBS) and allergies. Crohn's disease and ulcerative colitis, the major forms of IBD in humans are associated with exaggerated and poorly controlled Th1 or Th2 responses, respectively, and with a more complex networks of cytokine interactions, involving Th17 [3].

Lactobacilli have been shown to activate monocytes and macrophages, which play a pivotal role in antigen processing, presentation and activation of antigen-specific immunity and to stimulate IgA immunity. In particular, these cells together with dendritic and T regulatory cells are essential in the deviation of immune response to the so called type 1 response with cytotoxic effector cells or towards type 2 response characterized by antibody response. Type 2 response is related to secretion of IL-4, IL-5, IL-9 and IL-13 which promote induction of IgE and allergic response.

Effects of lactobacilli on host immune systems are known to depend on the bacterial species involved, since different strains are able to stimulate release of different cytokines. Results pointing toward stimulation of both Th1 and Th2 responses have been observed in animals fed with probiotics while few or no data are available on strains of human origin [4].

Aim of this study was to in vitro evaluate the release of pro- and anti-inflammatory cytokines induced by four strains of *Lactobacillus salivarius *of human origin.

### Bacterial strains and cell cultures

*Lactobacillus salivarius *strains LDR0723, BNL1059, RGS1746 and CRL1528 were isolated from vaginal brushing or faeces of four different apparently healthy subjects, who declare to have not consumed probiotic containing products in the two weeks preceeding sample collection. Samples were plated on homofermentative-heterofermentative differential (HHD) agar and incubated for 48-72 h in anaerobiosis.

Lactobacilli strains were initially identified by means of a biochemical assay based on carbohydrate fermentation (API50 CHL, BioMerieux Marcy L'Etoile, France). Identification of *L. salivarius *strains was further confirmed by PCR, as described by Chaugnaud et al [5].

Lactobacilli were stored at -80°C in MRS broth supplemented with 10% of glycerol until use.

Before experiments, bacteria were thawed and grown on MRS agar plates at 37°C in 10% CO_2 _enriched atmosphere for 24 h for two times and then subcultured in MRS broth for 24 h at 37°C in 10% CO_2 _enriched atmosphere.

The human macrophage-like cell line THP-1 (Istituto Zooprofilattico Brescia, Italy) was grown in culture flasks in RPMI-1640 medium (Sigma - Aldrich, Milan, Italy) enriched with 10% heat-inactivated foetal bovine serum (Sigma-Aldrich), 0.05 mM β-mercaptoethanol (Sigma-Aldrich), 1% Na-pyruvate (Sigma-Aldrich), 1% glutamine (Sigma-Aldrich) and 1% penicillin/streptomycin (Sigma-Aldrich). Cells were incubated at 37°C in a humified atmosphere containing 5% CO_2_.

### Stimulation of THP-1 cells with lactobacilli

Overnight bacterial cultures were washed twice with phosphate buffered saline buffer (PBS), pH 7.2, before being resuspended at a concentration of about 2 × 10^9 ^CFU/ml

Ten microliters of each bacterial suspension were transferred into the wells of a 24-wells plate containing 2 × 10^6 ^THP-1 cells/mL, and incubated at 37°C in a 5% CO_2_/95% air atmosphere. After 24 h incubation, the supernatant was aspirated, centrifuged at 2000 rpm and stored at -20°C. Cells receiving PBS only were used as unstimulated control. Each test was performed in triplicate. Vitality of *L. salivarius *strains, evaluated by means of growth curves, was not affected by incubation in RPMI-1640 medium. No notable changes in THP-1 cells counts were observed after incubation with *L. salivarius *with respect cells receiving only PBS.

Production of IL-12, IFN-γ, IL-4 and IL-5 was determined by means of specific quantitative enzyme-linked immunosorbent assays (ELISA) (BenderMedSystems, Wien, A), according to the manufacturer's instructions. Absorbance was measured at 450 nm using a Biorad spectrophotometer (mod 680 Biorad, Segrate, Italy). For each cytokine a standard curve was constructed in duplicate and used to quantify amount of cytokine (pg) per mL of culture medium. Ratio between Th1 and Th2 response was calculated as ratio between concentrations of Th1 cytokines (IL-12 + IFN-γ) and Th2 interleukins (IL-4 + IL-5).

Results were reported as differences (%) vs unstimulated cells of three different experiments in triplicate. Statistical differences were evaluated with one way ANOVA followed by Tuckey T test. Differences were considered as statistically significant when P-value was equal or less than 0.05.

**Figure 1:**
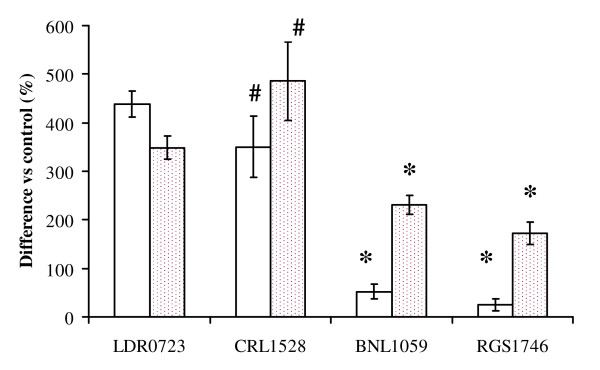
**Release of Th1 cytokines by THP-1 cells**. Open bars: IFN-γ, Pointed bars: IL-12. Data are means of three experiments ± SD *p < 0.05 vs LDR0723 and CRL1528; #: p < 0.05 vs LDR0723.

The four strains of *L. salivarius *showed a different ability to modulate production of IFN-γ and IL-12 in THP-1 monocyte cells (Figure 1). Production of IFN-γ was greatly stimulated by strains LDR0723 and CRL1528, while only slight increments were observed in cells treated with BNL1059 and RGS1746. Significant differences in IFN-γ production were observed between LDR0723 and BNL1059 and RGS1746 and between CRL1528 and BNL1059 and RGS1746. All the four strains stimulate production of IL-12, although at different rate, with LDR0723 and CRL1528 inducing significantly higher levels than BNL1059 and RGS1746.

**Figure 2:**
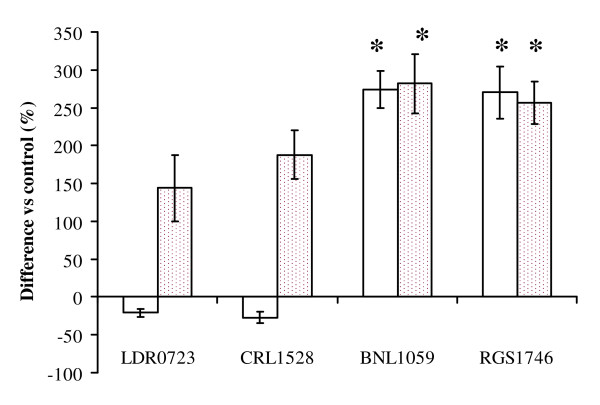
**Release of Th2 cytokines by THP-1 cells**. Open bars:IL-4, Pointed bars: IL-5. *p < 0.05 vs control (unstimulated cells). Data are means of three experiments ± SD *p < 0.05 vs LDR0723 and CRL1528.

With respect to unstimulated cells, BNL1059 and RGS1746 led to an increment in production of IL-4 in THP-1 cells, while diminished levels of IL-4 were observed in LDR0723 and CRL1528 treated cells (Figure 2). Production of IL-5 was stimulated by all the four strains, with significant higher levels observed in BNL1059 and RGS1746 treated cells with respect to LDR0723 and CRL1528 strains (Figure 2).

**Figure 3:**
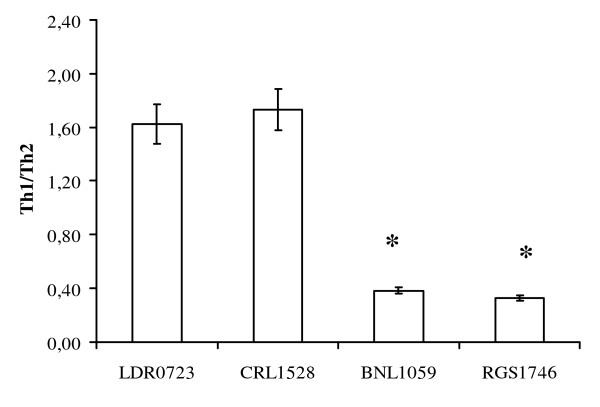
**Ratio between Th1 and Th2 responses**. Data are means of three experiments ± SD *p < 0.05 vs LDR0723 and CRL1528.

Th1/Th2 ratios are shown in Figure 3. *L. salivarius *strains LDR0723 and CRL1528 significantly moved Th1/Th2 balance toward Th1 response, while BNL1059 and RGS1746 led to a decrease of the Th1/Th2 ratio in favour of pro-inflammatory cytokines.

## Conclusions

Inflammation and immunity changes are generally critical for the development of nearly every complex condition. Alterations in equilibrium between Th-1 and Th-2 responses have been linked to the pathogenesis of a wide variety of diseases such as IBD, IBS and allergies. Different patterns of cytokines are involved in the pathogenesis of these diseases and they may act in opposite ways, as observed, for instance in IBD, where Crohn's disease lesion is related to a predominant activation of Th1-lymphocytes, while lesion of ulcerative colitis is mainly driven by Th2 cytokines [6,7]. Knowledge on the immune-mediated mechanisms in metabolic scenario has markedly increased in the recent past, evidencing the role that dietary components may have to modulate immunity by enhancing or suppressing the immune response. For instance, certain strains of probiotics have been demonstrated to be able to modulate the immune system by stimulating release of different patterns of cytokines by different cells [8].

In this study, four strains of *L. salivarius *of human origin were evaluated for their capabilities to influence production of pro- and anti-inflammatory cytokines by THP-1 cells. These cells were preferred to peripheral blood monocytes (PBMC) since the use of a cell line allowed us to standardize the assay, avoiding variability due to the fact that PBMCs harvested by apparently healthy subjects, may be influenced in their response by intrinsic factors due to individual variability.

Our results showed that the observed effects on cytokines' release are strictly strain-specific, since strains of the same species may modulate different patterns of cytokines and thus they may have opposite effects in the same condition. In fact, of the four strains of *L. salivarius *tested in the study, two modulated cellular immunity towards Th1, while the rest favoured a Th2 response. In comparison to unstimulated cells, LDR0723 and CRL1528 strains seemed to differ in their activity. In fact, LDR0723 favoured production of IL-12 rather than of IFN-γ, while a more sustained increment in release of IFN-γ than of IL-12 was produced by CRL1528. An increment in production of IL-12 levels was found also in cells stimulated with BNL1059 and RGS1746, even though they mainly oriented Th1/Th2 balance vs Th2. IL-12 has an essential role in Th1 development, stimulating activated T cells and NK cells to maximally produce IFN-γ, and inhibiting IL-4 induced IgE production by human PBMC *in vitro *[9]. In turn, IFN-γ promotes differentiation of naive Th lymphocytes (Th-0) towards a Th-1 subsets, prevents proliferation of Th2 lymphocytes and acts on B cells to inhibit switching to IgE [10]. By contrast, BNL1059 and RGS1746 favoured production of IL-4 which is known to promote production of IgE [10] and, at a lesser extent, of IL-5 which specifically controls production and activation and localization of eosinophils, the major cause of tissue damage in atopic disease [11]. Eosinophils have been recently shown to constitutively store cytokine associated with Th1, Th2 and T regulatory responses, although a preferential Th2 cytokine secretion occurs in response to immuno-polarizing cytokine signal [12]. Lack of IL-4 has been recently reported to modulate gut mucosal response in food allergy through diminished expression of TNF-α mRNA, increased Th1 IFN-γ, IL-12p40 regulatory cytokines, thus demonstrating their relevance in the control of allergic inflammatory processes, especially in the intestine [13]. Moreover, IL-12, IFN-γ and IL-4 inhibit Th17 differentiation for human cells, which is the main responsible rather than Th1 subsets for inflammation in autoimmunity disorders [14,15]. IL-4 and IL-5 are required to promote maturation of IgA, which appears, in terms of humoral immunity at mucosal surfaces, to combine properties of a neutralizing agent and of a mucosal immunopotentiator inducing effector immune response in a noninflammatory context favourable to preserve local homeostasis of the gastrointestinal tract [2].

The mechanism involved in the different immuno-stimulatory ability of different strains of lactobacilli belonging to the same species has not yet been fully elucidated and its evaluation was beyond the aim of this study. The differential immunostimulating property of LTAs from bacteria with different pathogenicity seems to correlate with content of D-alanine residues in the repetitious chains of the LTA. In addition, a mutant of *Lactobacillus plantarum *defective in D-alanylation of LTA has been shown to reduce the release of inflammatory cytokines from PBMC [16,17]. Therefore, composition in LTA might influence release of inflammatory cytokines [16,17] as well as it has been shown that the strain-dependent ability to promote synthesis of IL-12 may arise, at least in part, from the amount of peptidoglycan present in gram-positive bacteria, and that integrity of bacterial cell wall is essential for the induction of IL-12 production [18,19]. Moreover it may be hypothesized that other cellular components or bacterial products released by each single strain might be involved.

In the present study only four cytokines were assessed. However, they are representative of Th1 or Th2 responses and have been often used to evaluate the effect of lactobacilli and other bacteria on immune system [20-22].

In conclusion data obtained in this study evidenced that strains of the same species of *Lactobacillus *exhibited different modulation of immune response towards Th1 or Th2. For this reason, assessment of candidate probiotic strains for their immunomodulatory properties seems to be of notable importance to assure safety of their use and efficacy in treatment of immune-mediated diseases.

## Competing interests

The authors declare that they have no competing interests.

## Authors' contributions

LD participated in designing the study, data analysis and in the writing of the paper. LN performed all experiments and participated in data collection and analysis. EI participated in writing of the paper. EDV participated in designing the study, data analysis and in the writing of the paper. GB contributed to preparation of the manuscript. All authors read and approved the final manuscript.
